# The impact of the leg-lengthening total hip arthroplasty on the coronal alignment of the spine

**DOI:** 10.1186/1748-7161-10-S1-P12

**Published:** 2015-01-19

**Authors:** Yuichiro Abe, Shigenobu Sato, Satomi Abe, Takeshi Masuda, Kentaro Yamada

**Affiliations:** 1 Eniwa Hospital, Japan

## Objective

Coronal imbalance of pelvis is recognized to develop a degenerative lumbar scoliosis. We hypothesized that an abrupt change of pelvic obliquity may show a reproducible trend of coronal compensation in the lumbosacral spine. The aim of the study was to classify the change of coronal alignment of spine after THA.

## Materials and methods

This is a retrospective study based on the radiological analysis of 195 patients who underwent THA between 2009 and 2010. The mean age at surgery was 61.5 years old, and minimum follow up period was 24 months. Pelvic obliquity (POb), Cobb's angle of lumbar scoliosis (LS), and C7 plumb line in coronal plane were measured. Over 3.5 degrees of change in POb was regarded as delta-POb(+) and over 10 degrees of lumbar scoliosis was regarded as LS(+). The change of LS were classified into 3 subtypes; delta-LS(+), over 5degrees of progress in LS, delta-LS(-), over 5degrees of improvement in LS, and delta-LS(n), changes in LS within 5 degrees.

## Results

Over 3.5 degrees of change in POb was significantly correlated with the change in LS. In all 195 patients, 120 patients showed the improve of pelvic obliquity (delta-POb(+)). 99 patients in the 120 delta-POb(+) patients did not showed change (54, delta-LS(n)) or the improve of scoliosis (45, delta-LS(-)). No progression of lumbar scoliosis was observed in 75 delta-POb(-) patients. Remained 21 patients showed the progress or development of de novo scoliosis. Cases who failed to compensate the POb change at lumbosacral area developed de novo lumbar scoliosis (7 cases), showed progression of lumbar scoliosis (7 cases) or developed coronal trunk shift over 20mm (7 cases).

## Discussion

The patterns of compensation in lumbar or lumbosacral spine in coronal plane after leg lengthening THA were classified as regards to pelvic obliquity and cobb angle. 89.2% of all 195 patients showed acceptable compensation in lumbar spine, 21 patients developed coronal imbalance and 2 patient developed painful scoliosis. THA therefore considered to safe as regards to spinal balance in coronal plane. However we keep in mind that preoperative rigid scoliosis could have a risk for progress spinal imbalance.

